# Platelet-Rich Plasma as a Potential New Strategy in the Endometrium Treatment in Assisted Reproductive Technology

**DOI:** 10.3389/fendo.2021.707584

**Published:** 2021-10-18

**Authors:** Yunying Lin, Jia Qi, Yun Sun

**Affiliations:** ^1^ Center for Reproductive Medicine, Renji Hospital, School of Medicine, Shanghai Jiao Tong University, Shanghai, China; ^2^ Shanghai Key Laboratory for Assisted Reproduction and Reproductive Genetics, Shanghai, China

**Keywords:** platelet-rich plasma, endometrial receptivity, mechanisms, assisted reproduction techniques, risk

## Abstract

The success rate of assisted reproduction techniques (ART) has long been less than satisfactory albeit the great progress made in recent years, demonstrating the need for alternative options in the ART cycles. Growing evidence correlates the effect of intrauterine platelet-rich plasma (PRP) infusion on the endometrium with reassuring reproductive results. Thus, in this review, we focus on the current clinical and mechanical evidence on PRP and its effect on endometrial receptivity, and assess the features, benefits and limitations of the current studies and potential risks of PRP in ART.

## Introduction

Successful implantation requires a receptive endometrium, a functional embryo and a synchronized interaction between blastocyst and endometrium (1). Though advances in assisted reproduction techniques (ART) have led to remarkable improvements in pregnancy rates, implantation failure has long been an unsolved problem. One-third of implantation failures can be attributed to embryo reasons, while unsatisfactory endometrial receptivity and poor embryo-endometrium communication account for the remaining two-thirds of such failures (1). Despite the great progress in embryo election and quality improvement, many patients are still undergoing repeated implantation failure (RIF). Poor endometrial receptivity has become a bottleneck issue in the ART field, and the lack of evidence-based treatments signifies the issue. Clinicians and scientists have struggled to find an effective therapeutic solution.

Recently, increasing evidence has shown the positive role played by autologous platelet-rich plasma (PRP) in treating endometrium (2, 3). PRP is a volume of plasma, obtained by centrifugation of the patient’s whole blood, that has a platelet count above baseline (2, 4). With the intrauterine infusion of PRP, numerous proteins, several growth factors (GFs), and cytokines stored in the platelet act on the endometrium through the promotion of cell proliferation and neoangiogenesis, and the anti-inflammatory properties, resulting in successful implantation (2).

PRP treatment is gaining researchers’ attention due to its unique advantages. As an autologous biologic material, PRP minimizes the risk of immune reactions and contagious diseases (5). In addition, the application of PRP is considered less invasive since it is produced from peripheral blood (6).

In this article, we review PRP and its application to clinical medicine, with an emphasis on reproduction. We then address the current clinical and mechanical evidence for the influence of PRP on endometrial receptivity. Last but not least, we analyze the limitations of the current studies and potential risks of PRP in co-treatment in ART.

## PRP and its Clinical Application

PRP can be defined as a volume of plasma that has a platelet count above baseline prepared by centrifugation of the patient’s peripheral blood (2, 4). Platelets are non-nucleated cell fragments evolved from bone marrow-derived megakaryocytes. The cytoplasm of platelets is comprised of two parts: the granules accumulated chromomere and the agranular hyalomere rich in cytoskeletal proteins. Platelet granules contain multiple proteins including antimicrobial peptides (7), fibronectin and vitronectin (8), GFs [including platelet-derived growth factor (PDGF), the epidermal growth factor (EGF), the transforming growth factor β-I (TGFβ-I), the vascular endothelial growth factor (VEGF), the hepatocyte growth factor (HGF), the basic fibroblast growth factor (bFGF) (9)], and cytokines [comprising anti-inflammatory and pro-inflammatory interleukins, interleukin-4 (IL-4), IL-8, IL-13, IL-17, tumor necrosis factor-α (TNF-α), and interferon (IFN)-α (4)]. These proteins are secreted in the event of injuries so that platelets are activated and then delivered to the site of injury or deficiency (4, 8), allowing PRP to participate in cell proliferation, migration, tissue growth and healing, neoangiogenesis (2), inflammatory processes (7, 10), chemotaxis and immune responses (3).

Platelet concentrates can be sorted into four categories based on their leucocyte and fibrin content: pure platelet-rich plasma (P-PRP), leucocyte- and platelet-rich plasma (L-PRP), pure platelet-rich fibrin (P-PRF), and leucocyte and platelet-rich fibrin (L-PRF) (11). Each one has a particular bioaction and has been applied in a specific clinical field. However, the overall goal is to achieve a concentrating platelet, as well as factors of two to three times in whole blood, in order to make PRP a more effective instrument than peripheral blood in the processes described above (2). Factors in platelet granules continue exocytosing after their first-round release at activation, maintaining their levels three- to five-fold higher as compared to baseline values (12).

Applications of PRP are commonly disciplined in various conditions related to regenerative medicine without known effective treatments, such as osteoarthritis (13) and ligament injuries (14) in orthopedics, skin rejuvenation and hair loss in dermatology (15) and breast augmentation and wound healing in aesthetic surgery (16). Latest reports also record the application of PRP in reproduction: PRP has been found to facilitate growth rate and cell viability of primordial or primary to pre-antral stage follicles, and improve ovarian function, pregnancy and birth rate (17–20). In this article, we will review and summarize the preparation procedure of PRP, the function of PRP on endometrium and its contribution to the improvement of reproductive results.

## PRP Preparation

Most studies share similar PRP preparation procedures drawing patients’ own venous blood aiming at obtaining a three-layer separation from the whole blood first, which contains the cellular plasma in supernatant, the intermediate buffy coat containing concentrated platelets, and the bottom red blood cells, and then mixing the pellet of platelets with 0.5-1ml of supernatant to get the final PRP sample. However, multiple inconsistent details in various studies may potentially lead to contradictory outcomes. For example, in the relevant articles, the amount of venous blood drawn from the patient varied from 8ml to 18ml, and the relative centrifugal force differed from 300*g for 10min to 3300*g for 7min. Therefore, different concentrations of platelets and leukocytes could be obtained, which may in turn impact the amount of various types of growth factors playing a key part in the PRP samples. Still, researchers haven’t reached a consensus on whether to use the activated or inactivated platelets, as well as the type of the platelet agonists for activation, including calcium chloride, thrombin, etc. (21, 22). Only a minority is centrifuged again in order to remove cellular debris through a filter (21). Besides, few paid attention to the temperature when processing the PRP. Since the standard PRP preparation protocol is the prerequisite of the evaluation of different results in various trials, it is crucial to make every attempt to develop the optimal protocol for PRP preparation.

## Clinical Evidence of PRP on Endometrial Receptivity

Research in the effect of PRP on the endometrium has surged in recent five years. Most studies apply PRP in clinical cases of patients with thin endometrium, repeated implantation failure (RIF), chronic endometritis (CE) and Asherman Syndrome (AS), since unfortunately, there has not yet been any effective remedy for these distressing problems. It has been demonstrated that intrauterine PRP infusion positively influences the reproductive results, including endometrial thickness, clinical pregnancy, live birth, etc., thus can be potentially included in the different protocols for endometrial preparation. **Tables 1**–**3** outline the main characteristics of almost all related and eligible studies, among which five studies are randomized clinical trials (RCTs), and one being a single arm RCT. The studies cover different populations, including patients with thin endometrium (<7mm) in 8 studies, patients with RIF in 7 studies, and patients with refractory endometrium and suboptimal endometrial growth. No consensus has been reached on the optimal timing of PRP injection. For the patients with thin endometrium, the timing of PRP infusion varied from the 8^th^ day to the 17^th^ day of HRT cycle, and for those who underwent RIF, PRP therapy was usually applied 2 days before the embryo transfer (ET). Seventeen out of eighteen studies administered the PRP with the dose of 0.5-1.0 ml, the remaining one with a dose <0.5ml. We will now analyze the related studies of intrauterine PRP infusion published in various clinical ART settings.

**Table 1:** Studies assessing the effect of platelet-rich plasma (PRP) treatment in patients with thin endometrium *in vitro* fertilization.

Year of Publication	Country	Year of Study	RCT	Objects	Sample Size		Intervention	Control	Time of PRP Infusion	Transfer Type	Outcome Measures		Effects				Reference
					Case	Control							Case (After PRP treatment)	Control (Before PRP treatment)	P Value*		
2015	China	March 2014 to June 2014	No	Women aged 31-39 with thin endometrium (<7 mm) after standard HRT	5	/	HRT+Intrauterine infusion of 0.5-1 ml of PRP	/	On the 10th day of HRT cycle (another dose of infusion of PRP was perfomed 72h later if the endometrial thickness was not satisfied)	FET	Endometrial thickness	Reached 7mm with one or two doses	5/5	0/5	/		(23)
											Clinical pregnancy		5/5	0/5	/		
											Live birth		4/5	0/5	/		
2017	Iran	September 2015 to May 2016	No	Women with a history of inadequate endometrial growth (<7 mm) in FET	10	/	HRT+Intrauterine infusion of 0.5ml PRP	/	On the 11-12th day and repeated on 13-14th day of HRT cycle	FET	Endometrial thickness	Reached 7mm with one dose	0/10	0/10	/		(24)
												Reached 7mm with two infusions	10/10	0/10	/		
											Chemical pregnancy		5/10	0/10	/		
											Clinical pregnancy		4/10	0/10	/		
2018	Iran	September 2016 to January 2017	Yes	Women aged 18-42 with thin endometrium (<7 mm)	40	43	HRT+Intrauterine infusion of 0.5-1.0ml of PRP	Underwent ET without intrauterine administration	On the 13th day of HRT cycle	FET	Endometrial thickness (mm)		8.67 ± 0.64	8.04 ± 0.27	0.001	↑	(25)
											Clinical pregnancy (per-cycle)		13/40 (32.5%)	6/43 (14.0%)	0.044	↑	
											Implantation rate		21%	9.37%	0.002	↑	
											Ongoing pregnancy (per-cycle)		11/40 (27.0%)	6/43 (14.0%)	0.127	(–)	
2019	Iran	2016 to 2017	Yes	Women who had a history of canceled frozen-thawed embryo transfer cycle due to a thin endometrium (<7 mm)	30	30	HRT+Intrauterine infusion of 0.5ml of PRP	Sham catheter	On day 11-12 and was repeated after 48 h	FET	Endometrial thickness	One dose	5.993 ± 0.701	5.453 ± 0.823	0.63	(–)	(26)
												Two doses	7.213 ± 0.188	5.767 ± 0.973	< 0.001	↑	
											Chemical pregnancy		12/30(40%)	2/30(6.7%)	0.031	↑	
											Clinical pregnancy		10/30(33.3%)	1/30 (3.3%)	0.048	↑	
2019	China	July 2015 to July 2016	No	Women aged under 40 with thin endometrium (<7 mm)	34	30	HRT+Intrauterine infusion of 0.5-1.0ml of PRP	Underwent ET without intrauterine administration	On the 10th day of HRT cycle	FET	Endometrial thickness (mm)		7.65 ± 0.22	6.52 ± 0.31	0.013	↑	(22)
											Clinical pregnancy		15/34 (44.12%)	6/30(20%)	0.036	↑	
											Implantation rate		19/34(27.94%)	7/30 (11.67%)	0.018	↑	
2019	South Korea	December 2015 to June 2017	No	Women age of 20–45 years who had a history of two or more failed IVF cycles and refractory thin endometrium (<7mm)	20	/	HRT+Intrauterine infusion of 0.7-1.0ml of PRP	/	On day 10 and was repeated at 3 day intervals until the EMT reached 7 mm, 3 days before ET	FET	Endometrial thickness (mm)		6.0 ± 1.1	5.4 ± 0.8	0.070	(–)	(27)
											Clinical pregnancy		6/20	0/20	0.020	↑	
											Live birth		4/20	0/20	0.106	(-)	
											Implantation rate		7/55	0/52	0.015	↑	
2020	India	July 2018 to September 2019	No	Women aged 27 to 39 years, suffering from primary or secondary infertility with thin endometrium (<7 mm)	32	/	HRT+ Instillation of 4ml PRP(1ml in each wall of the uterine cavity) into the endomyometrial junction	/	7–10 days after the injection of leuprolide (on day 16 of OCP), 22-27days before ET	FET	Endometrial thickness	≥7 mm (embryo transfer done)	24/32(75%)	0/32	/		(28)
												6–7 mm (embryo transfer not done)	4/32(12.5%)	0/32	/		
												<6 mm no improvement (embryo transfer not done)	4/32(12.5%)	0/32	/		
											Serumβ-hCG positive		12/24(50%)	0/24	/		
											Clinical pregnancy		10/24(41.66%)	0/24	/		
											Live birth		5/24(20.83%)	0/24	/		
2020	Brazil	January to December 2018	No	Patients with endometrium thickness below 5 mm during preparation for FET	21	/	HRT+Intrauterine infusion of 0.5ml of PRP	/	PRP was administered on the 14th to 17th day of HRT every second day, for a total of three infusions.	FET	Clinical pregnancy		0.667	0/21	/		(29)
											Ongoing pregnancy or live birth		0.54	0/21	/		

*: ↑, increase; (-), no significant change.

**Table 2:** Studies assessing the effect of platelet-rich plasma (PRP) treatment in repeated implantation failure (RIF) patients *in vitro* fertilization.

Year of Publication	Country	Year of Study	RCT	Objects	Sample Size		Intervention	Control	Time of PRP Infusion	Transfer Type	Outcome Measures	Effects				Reference
					Case	Control						Cases	Control	P Value*		
2016	Iran	March to June 2016	A single arm preliminary study of an RCT	Women aged under 40 who failed to conceive after 3 or more ET with high-quality embryos	20	/	HRT+Intrauterine infusion of 0.5ml of PRP	/	48h before ET	FET	Clinical pregnancy	18/20(90%)	0/20	/		(30)
											Ongoing pregnancy	16/20(80%)	0/20	/		
											Molar pregnancy	1/20(5%)	0/20	/		
2019	Turkey	January 2014 to January 2017	No	Patients aged between 21 and 39 with a history of at least three consecutive failed IVF	34	36	HRT+Intrauterine infusion of 1ml of PRP	Underwent ET without intrauterine administration	48h before ET	FET	Endometrial thickness (mm)	10/(8–14)	6.25(4.3–6.9)	<0.001	↑	(31)
											Clinical pregnancy	17/34(50%)	12/36(33.3%)	0.042	↑	
											Live birth	14/34(41.2%)	6/36(16.7%)	0.045	↑	
2019	Iran	2016-2017	No	Patients with history of more than 2 repeated failed embryo transfer cycles	67	56	HRT+Intrauterine infusion of 1ml of PRP	Underwent ET with systemic administration of GCSF	48h before ET	FET	Chemical pregnancy	29/67 (43.3%)	15/56 (26.8%)	0.057	(-)	(32)
											Clinical pregnancy	27/67(40.3%)	12/56 (21.4%)	0.025	↑	
2019	Brazil	February 2017 to October 2017	No	Patients with ≥2embryo transfers, and at least 5 good-morphological embryos were transferred	33	33	HRT+Intrauterine infusion of 0.7ml of PRP+subcutaneous G-CSF injection (300mg/0.5ml started simultaneously to PRP and was administered subcutaneously every week)	Patients in their first IVF/ICSI cycle attempt without PRP or G-CSF treatment	48h before ET	ICSI	Clinical pregnancy	12/33(36.4%)	10/33(30.3%)	0.61	(-)	(33)
											Implantation rate	14/77(18.2%)	12/68(17.6%)	0.90	(-)	
											Miscarriage rate	3/12(25.0%)	1/10(9.0%)	0.43	(-)	
											Ongoing pregnancy	9/33(27.3%)	9/33(27.3%)	0.99	(-)	
											Live birth	9/33(27.3%)	9/33(27.3%)	0.99	(-)	
2020	Iran	2016-2017	Yes	Patients aged below 40 years with history of 3 or more embryo transfer failures with high-quality embryos and candidates for FET	49	48	HRT+Intrauterine infusion of 0.5 ml of PRP	Underwent ET without intrauterine administration	48h before ET	FET	Chemical pregnancy	26/49(53.06)	13/48(27.08)	0.009	↑	(34)
											Clinical pregnancy	22/49(44.89%)	8/48(16.66%)	0.003	↑	
2020	Iran	2016-2018	No	Women aged under 35 with a failure to achieve a clinical pregnancy after the transfer of at least four good-quality embryos in at least three fresh or frozen cycles and normal endometrial thickness (≥7 mm)	42	43	HRT+Intrauterine infusion of 1ml of PRP	Underwent ET without intrauterine administration	48h before ET	FET	Biochemical pregnancy	15/42(35.7%)	16/43(37.2%)	0.89	(-)	(35)
											Clinical pregnancy	13/42(31.0%)	16/43(37.2%)	0.54	(-)	
											Ongoing pregnancy	11/42(26.8%)	11/43(25.6%)	0.90	(-)	
2020	Iran	2016-2019	Yes	Women aged between 20–40years who failed to be pregnant after three or more embryo transfer of embryos with good quality	55	43	HRT+Intrauterine infusion of 0.5ml of PRP	Underwent ET without intrauterine administration	48h before ET	FET	Clinical pregnancy	29/55(52.7%)	10/43(23.3%)	0.001	↑	(36)
											Ongoing pregnancy	28/55(50.9%)	7/43(16.3%)	0.001	↑	
											Implantation rate	35/55 (63.6%)	15/43 (34.9%)	0.001	↑	

*: ↑, increase; (-), no significant change.

**Table 3:** Studies assessing the effect of platelet-rich plasma (PRP) treatment in other patients *in vitro* fertilization.

Year of Publication	Country	Year of Study	RCT	Objects	Sample Size		Intervention	Control	Time of PRP Infusion	Transfer Type	Outcome Measures	Effects				Reference
					Case	Control						Cases/After PRP treatment	Control/Before PRP treatment	P Value*		
2017	India	January to August 2016	No	Women aged 20-40 with suboptimal endometrial growth (endometrium thickness < 7mm despite standard dose of estradiol valerate (up to 16mg/day), or suboptimal endometrial vascularity (<5 vascular signals reaching the central zone (zones 3 and 4 as per Applebaum grading) of the endometrium), and repeated cycle cancellations	64	/	HRT+Intrauterine infusion of 0.5-0.8ml of PRP	/	On the 15th or 16th day of HRT cycle, and another dose 72h later if necessary	FET	Endometrial thickness (mm)	7.22	5	< 0.00001	↑	(37)
											Vascularity	sparse, modest → excellent vascularity pattern (17) sparse → modest vascularity pattern (52) persistant sparse vascularity pattern (4)		/		
											Serumβ-hCG positive	39/64(60.93%)	0/64	/		
											Clinical pregnancy	29/64(45.31%)	0/64	/		
2018	China	January to October 2017	No	Women aged 27-43 who underwent RIF with thin endometrium (<7 mm) or suboptimal endometrial vascularity with <5 vascular signals reaching the central zone (zones 3 and 4 as per Applebaum grading)	20	/	HRT+Intrauterine infusion of 0.5-0.8ml of PRP	/	Not mentioned	FET	Endometrial thickness	7.82 ± 1.04	5.55 ± 0.71	<0.0001	↑	(21)
											Pregnancy rate	12/20(60%)	0/20	/		
2018	Venezuela	February 2016 to February 2017	No	Women aged 33-45 with a history of refractory endometrium (characterized by atrophy with endometrial interface measurements below 6 mm by ultrasound; and/or the Asherman syndrome) and and at least one failed IVF attempt	19	/	HRT+Intrauterine infusion of 1ml of PRP	/	On the 10th day of HRT cycle, and then 72 hours after the first administration.	IVF-ET	Serumβ-hCG positive	14/19(73.7%)	0/19	/		(38)
											Live birth	5/19(26.3%)	0/19	/		
											Ongoing pregnancy	5/19(26.3%)	0/19	/		
											Chemical pregnancy	2/19(10.5%)	0/19	/		
											Anembryonic pregnancies	1/19(5.3%)	0/19	/		
											Endometrial thickness	<7mm (with one dose);<9mm(with two doses)	Not mentioned	/		
2018	India	January 2018 to November 2018	No	Women aged 25-40 who had at least one previous FET failure with endometrium 7 mm or more in thickness	42	56	HRT+Intrauterine infusion of 0.3-0.4ml of PRP	Underwent ET without intrauterine administration	On the 8th or 9th day of HRT cycle	FET	Clinical pregnancy	47.6%	42.8%	0.09	(-)	(39)

*: ↑, increase; (-), no significant change.

### Thin Endometrium

Endometrium is one of the most prominent factors in implantation and pregnancy. Multiple studies suggest that pregnancy rate may increase with growing endometrial thickness within a certain range (40–43). It has been suggested that the minimal endometrial thickness required for embryo transfer is 7 mm at the end of follicular phase (44, 45). Thus, endometrial thickness less than 7 mm is considered to be thin, which occurs in 2.4% of the *in vitro* fertilization cycles and is associated with a lower possibility of pregnancy (44, 46).

Six studies on PRP intrauterine infusion treatment of infertile women with thin endometrium were conducted in frozen embryo transfer (FET) cycles on the 10^th^ to 13^th^ day of hormone replacement therapy (HRT) cycle, reaching a consensus on the significant improvement in the pregnancy rate and the endometrial thickness with the PRP treatment (**Table 1**). Three articles displayed 100% otherwise infertile cases reaching endometrial thickness of 7mm with one or two times of PRP infusion (23, 24, 26), and another 2 revealed PRP infusion leading to significantly increased endometrial thickness compared with the controls (22, 25). The study in Brazil also showed reassuring clinical pregnancy, ongoing pregnancy or live birth rates after the PRP treatment, but did not mention the endometrial thickness improvement (29). A study in South Korea found no statistically significant difference in the endometrium thickness and the live birth rates between the PRP and control groups but there was a significant statistical difference in implantation and clinical pregnancy rates between the PRP group and the controls (12.7 and 30%, respectively) (27). The largest RCT study by Eftekhar et al. (25) was conducted on 83 infertile women, including 40 patients with 0.5-1.0ml intrauterine PRP infusion on the 13^th^ day of HRT cycle and 43 controls who underwent embryo transfer without intrauterine administration. The endometrium of the patients with PRP treatment expanded significantly to 8.67 ± 0.64 (from 6.09 ± 0.47) while the endometrium thickness of the controls merely increased to 8.04 ± 0.27 (from 6.15 ± 0.37) (p=0.001). The per-cycle clinical pregnancy rate raised from 14% in controls to 32.5% in the PRP group (p=0.044). However, there is no statistically significant difference in ongoing pregnancy rate between the two groups.

A recent research on 32 women suffering from primary or secondary infertility with thin endometrium (<7 mm) concluded that the injection of PRP (1ml in each wall of the uterine cavity) guided by hysteroscopy into the endomyometrial junction. In this study, PRP injection improved endometrial thickness and pregnancy rates in cases of previously canceled embryo transfer due to a thin endometrium (28). All studies have led to the conclusion that infertile women with thin endometrium benefit from PRP treatment, whereas the experimental conditions should be noticed and controlled, including PRP preparation process, PRP infusion dose, inclusion criteria etc.

### Repeated Implantation Failure

Infertile women with RIF represent another large potential group that could benefit from the PRP administration. The definition of RIF slightly differs based on various sets of standards. Most studies defined RIF as the failure of achieving a pregnancy after at least three embryo transfer cycles in which one or two high-grade embryos are transferred. Since the etiology of RIF has not been clinically confirmed, it is rather difficult to implement targeted therapy, leading to RIF patients being among the most difficult patients to treat, with no proven standard treatments.

Seven related studies, including 3 RCTs and 4 cohorts, were conducted, of which 6 studies detected encouraging results (**Table 2**). Two RCTs and a cohort study all implied significant improvements of clinical pregnancy rate with the use of intrauterine PRP infusion compared with controls, as well as other studied endpoints, including endometrial thickness, chemical pregnancy, implantation rate, ongoing pregnancy, and live birth rate. The RCT carried out in Iran with a population of 98 women with RIF indicated that intrauterine infusion of PRP increased the clinical pregnancy (intrauterine PRP infusion group *vs* control group: 48.3% *vs* 23.3%, p=0.001), ongoing pregnancy (intrauterine PRP infusion group *vs* control group: 46.7% *vs* 11.7%, p=0.001) and implantation rate (intrauterine PRP infusion group *vs* control group: 58.3% *vs* 25%; p=0.001) in PRP group compared with control group (36). Nazari et al. demonstrated similar results in their RCT, where increased clinical (intrauterine PRP infusion group *vs* control group: 44.89% *vs* 16.66%, p= 0.003) and chemical pregnancy rate (intrauterine PRP infusion group *vs* control group: 53.06% *vs* 27.08%) were observed in PRP group compared with control group (34). Mehrafza et al. compared the effect of intrauterine infusion of PRP with systemic administration of granulocyte colony stimulating factor(G-CSF) in a sample size of 123, and they demonstrated that intrauterine infusion of PRP could significantly enhance clinical pregnancy rate in RIF patients in comparison with systemic administration of G-CSF (32). One study demonstrated the efficacy of the combined use of intrauterine PRP and subcutaneous G-CSF (33). When compared with control patients in their first in vitro fertilization (IVF)/intracytoplasmic sperm injection (ICSI) cycle attempt without PRP or G-CSF treatment, no statistically significant difference regarding implantation, clinical pregnancy and miscarriage rates was found between the two groups, suggesting the combined use of intrauterine PRP and subcutaneous G-CSF a potential new strategy for endometrial receptivity improvement (33). However, another study holds opposing opinions. Tehraninejad et al. nonrandomly recruited 85 patients with RIF and normal endometrial thickness (≥7 mm), out of which 42 received 1ml PRP, and 43 were included in the control group. It is worth noting that there was no significant difference in the pregnancy outcomes between the two groups, including biochemical, clinical and ongoing pregnancy (≥20 weeks of gestation) rate (35). Till now, most studies on PRP and RIF confirm the positive role of PRP, but the efficacy of PRP on RIF patients remains controversial. The study design and the inclusion criteria should be controlled in order to investigate the effect of PRP.

### Chronic Endometritis

Chronic endometritis is a persistent inflammatory condition of the endometrial mucosa with plasmacyte infiltration in the endometrial stromal compartment caused by various bacterial pathogens (47, 48). In the endometrium of CE, the local expression of multiple genes, including chemokines, cytokines and apoptotic proteins, was dysregulated (49). Various reports indicate that the incidence of CE in infertile women who underwent IVF cycles is approximately 15%, and over 30% of the patients with RIF are diagnosed with CE, the presence of which is associated with overall lower success rates in ART (50–52). Since CE could modify the endometrial decidualization, it may affect the endometrial receptivity to accommodate pregnancy (53). Oral antimicrobial regimes are considered to be a traditional remedy for CE. Nevertheless, certain CE cases do not respond satisfactorily to these widely applied treatments such as doxycycline, ciprofloxacin and metronidazole (54), driving research to address the need for alternative options when managing these cases. Recent research suggested that PRP may be employed as an ideal CE treatment (55). The case report presents a woman diagnosed with CE with premature ovarian insufficiency and a history of six failed donated embryo transfers. Twin pregnancy and birth were finally achieved with 1.5ml intrauterine PRP infusion curing the CE, after the woman underwent another new failed ET with antibiotic treatment. Another case showed 5 infertile women with CE achieved endometrial histologically recovery and 100% positive serum β-HCG results with the application of intrauterine infusion of 0.5-0.8ml of PRP every 2 days for 3 cycles (56). Clearly more studies on larger patient population, as well as more RCTs, are needed for clinically meaningful conclusions.

### Asherman Syndrome

Asherman Syndrome (AS) consists of the development of intrauterine adhesions as a consequence of trauma, radiation, or infection in the endometrium. AS remains one of the most challenging pathologies fertility specialists encounter in daily practice. Prevalence of this syndrome varies from 2.8 to 45.5% in infertile women (57, 58).

There were only several cases of AS treated with intrauterine PRP infusion. Relevantly, it was reported that a 31-year-old women was diagnosed as AS with a postpartum curettage history (59). The patient received 1 ml autologous PRP on the 12^th^ day of HRT, and the procedure was repeated 3 days after, allowing endometrial tissue regeneration, with increased vascularity, endometrium thickness, and restoration of endometrial function that eventually led to a successful pregnancy. In a separate study, two women with AS experienced recurrent pregnancy loss after various treatments were reported (60). Both finally succeeded in pregnancy with PRP therapy, despite the persistent thin lining, indicating that PRP may not only help endometrial growth, but also enhance its functional properties. It can be interpreted that the removal of scar tissue, leading to the exposure of the normal endometrial cells to the GFs and cytokines in PRP, helps boost the existing cellular functions involved in tissue regeneration.

### Others

There are also some studies which contain different inclusion criteria described in detail in **Table 3**. All 4 studies take the patients with at least one instance of failed embryo transfer previously as the research object, among which 3 studies point out that the use of autologous PRP holds promise in all the studied results, including endometrium thickness and diverse pregnancy tests (21, 37, 38). Kaur et al. (39) collected data of 98 patients who had at least one previous FET failure with endometrium 7 mm or more in thickness and divided them into the intervention group with the treatment of intrauterine infusion of 0.3-0.4ml of PRP and the control group. The intervention failed to increase the clinical pregnancy rate in this study. A related meta-analysis pointed out that the administered PRP at a dose of 0.5–1 ml was more effective than those administered PRP at doses of ≤ 0.5 ml and ≥ 1 ml (3). Therefore, the dose of the PRP used may have a biased impact on the results.

## Potential Mechanisms of PRP on Endometrial Receptivity

**Figure 1:**
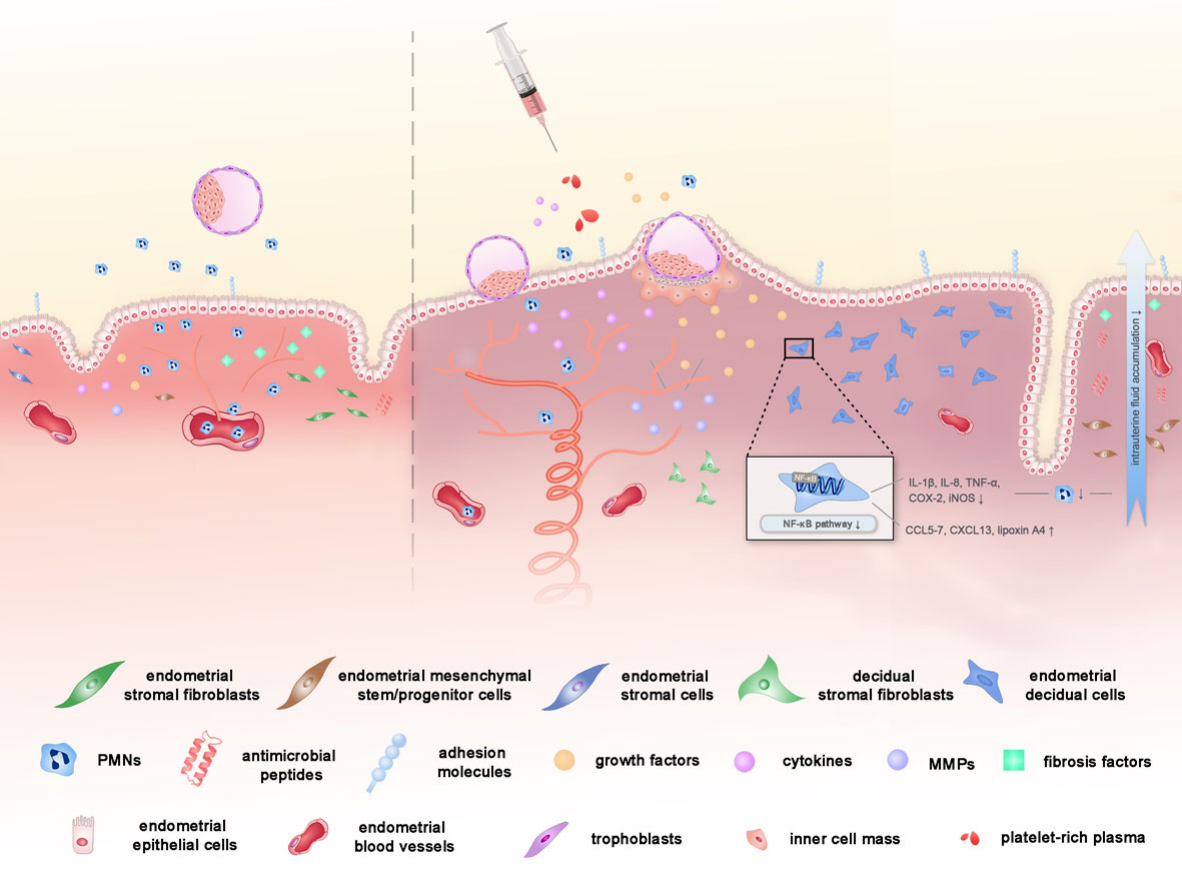
The potential mechanisms of PRP on endometrial receptivity in assisted reproductive technology. PRP might improve the endometrial receptivity through the improvement of cell proliferation, vascularization, anti-inflammatory properties and the reduction in the degree of fibrosis, with the help of the concentrated peptides, GFs and cytokines in PRP.

As growing evidence shows the correlation between satisfactory reproductive results and intrauterine PRP infusion treatment, researchers embark on the investigation of the underlying mechanisms of PRP in enhancing endometrial receptivity. Below are the potential mechanisms that account for the above clinical phenomena in the current literature (**Figure 1**).

First and foremost, PRP is known to play a critical role in the proliferation, regeneration and differentiation of cells due to its housing of a number of GFs, such as VEGF, PDGF, EGF, TGF, IGF1 and other cytokines, released upon platelet activation at the site of injury or inflammation (3, 61). Among the GFs, VEGF is responsible for vascularization as well. Evidence shows that PRP stimulates various endometrial cell proliferation including epithelial cells, endometrial stromal fibroblasts, and endometrial mesenchymal stem/progenitor cells (21, 61, 62). Addition of PRP significantly increased cell proliferation in human endometrial stromal cell line (ICE7), and the replication of the effects was observed when primary stromal cells were used. Murine model also showed that a significantly increased Ki67 expression in endometrium after PRP treatment (63). Gaining expression of adhesion molecules and attracted stem cells and increased endometrial cell migration with PRP infusion may in turn stimulate endometrial growth and receptivity (8, 61). Research showed that a higher level of Hoxa10, one of the primary endometrial markers of receptivity, was detected in the endometrium after the application of PRP (63). Therefore, PRP rich in GFs can be the potential solution to the infertility patients with thin endometrium, which has been pathophysiologically characterized by the state of high blood flow resistance and VEGF down-regulation with inadequate epithelial growth and vascularization (64–68).

Considering that tissue growth and cell proliferation depend not only on single GFs, but on an overall profitable environment, another potential explanation for the action of PRP on endometrial is *via* its anti-inflammatory and anti-microbial property (2). Therapeutic PRP works by inhibiting nuclear factor kappa-B (NF-κB) (69), a key valve of the inflammatory process, modulating prostaglandin-endoperoxide synthase 2 (COX-2) expression and other crucial inflammatory and pro-inflammatory cytokines, which have an important role in the implantation process (3). On one hand, PRP increases chemokines such as chemokine ligand 5 (CCL5), CCL7, CXC-motif chemokine ligand 13 (CXCL13) (61) and lipoxin A4 (69) that reduces the attraction of polymorphonuclear neutrophils (PMNs) to the inflamed tissue, through blood vessel walls into the uterine lumen (69), leading to decreased intrauterine fluid accumulation, thus alleviating the inflammation (69, 70). On the other hand, PRP poses a down regulation effect of cytokines, such as IL-1β, IL-8, TNF-α, COX-2 and inducible nitric oxide synthase (iNOS) expression *in vitro* and in animal models (62, 71). Additionally, the recorded presence of antimicrobial peptides in platelet secretory granules can also interpret the anti-inflammatory function of PRP (7). Since the endometrial environment should be switched to an anti-inflammatory state in the mid-secretory phase to prevent fetal rejection (72, 73), the mechanism of PRP treatment illustrated above may account for its reassuring clinical effects.

Last but not least, PRP treatment may have succeeded in reducing the degree of fibrosis *via* down-regulating expression of fibrosis-related factors including collagen type 1A (COL1A1), transforming growth factor β1 (TGFβ1), tissue inhibitor of metalloproteinase-1 (TIMP1), and finally enhanced implantation sites and live birth rate ensued (6, 71). Matrix metalloproteinase(MMP)1, MMP3, MMP7, and MMP26, members of MMP gene family, involved in tissue regeneration and wound healing *via* degradation of extracellular matrix (ECM) and wound remodeling (74), are activated by a wide range of cytokines and GFs present in PRP and then are elevated (62). Hence, in terms of intrauterine adhesions, where factors stimulating the formation of fibrotic tissue into the intrauterine environment are released, PRP may be a potential ideal cure due to the mechanism above (6).

Apart from the cases above, studies show that PRP synergizes with other treatments to enhance the endometrial regeneration. In a rat model of intrauterine adhesion (75), PRP amplifies the therapeutic effect of menstrual blood-derived stromal cell (MenSC), optimizing the pregnancy outcomes through the improvement of the epithelial thickness, vascularization, the decrease of inflammation and fibrosis with augmented soluble paracrine factors released by MenSCs including insulin-like Growth Factor-1 (IGF-1), stromal cell-derived factor-1 (SDF-1) and thrombospondin-1 (TSP-1), and suppression in inflammation by increasing IL-1β, IL-4, IL-10 and decreasing IL-6. The repairment derives from the stimulation of the Hippo signaling pathway with the regulation of the downstream factors connective tissue growth factor (CTGF), WNT5a and growth differentiation factor 5 (GDF-5). In another rat model of uterine horn damage (76), PRP therapy improves endometrial regeneration after BMSC transplant therapy, likely mediated through the NF-κB signaling pathway subunit p50 to directly induce the expression IL-10, and through the increase in endometrial thickness and angiogenesis.

## Limitations of the Current Research and Potential Risks of PRP Treatment in ART

Admittedly, both laboratory and clinical data make intrauterine PRP infusion a promising therapeutic approach in treating uterine endometrium. However, several limitations should be considered regarding the interpretation of the present studies and outcomes.

First, little is known about the best dosage and time or duration for the PRP treatment in endometrium. The importance of standard techniques and a highly repeatable protocol with sufficient description to prepare platelets for clinical use should be highlighted. Second, patients should be conferred upon separate treatment according to their putative cause of implantation failure in a more precise manner, so that the conclusion of the best beneficiary population of PRP treatment would be more convincing. Third, more rigid clinical trials, especially larger scaled RCTs, are warranted, even though they might be quite challenging to perform. Since intrauterine PRP infusion may result in local mechanical injuries which may affect endometrium receptivity, it is better to include sham catheter performance acting as a placebo, which is absent in most of the current studies, in the control group. However, due to its invasiveness and uncertain efficacy, it may well be that few patients are willing to make the attempt. Fourth, the indication of the PRP treatment should be considered more rigorous. As an autologous biologic material, PRP does appear to be considered relatively safe. However, some doubts remain on whether it is suitable for every infertile woman with thin endometrium, RIF and CE. Till now, there was only one study indicated that plasma from AS/endometrial atrophy patients would be comparable to plasma from healthy subjects (63). PRP primarily contains platelets, and some contains leucocytes as well. It is still not clear whether PRP extracted from the blood of patients with blood disorders like inflammation (CE, upper respiratory tract infection, etc.), leukemia and thrombocytopenia are different from that from healthy people, and for those patients, whether PRP therapy is still appliable and effective needs further discussion. Fifth, as PRP is a potential clinical application, more detailed mechanism is necessary. Last but not least, long-term health and complications of the resulting child, such as the high risk of endometrial cancer, should be considered. Due to PRP therapy being a novel application in reproductive medicine, it has been only 7 years since PRP infusion was first applied to endometrium. As reproductive results and endometrial thickness being the primary outcomes, follow-ups are basically terminated as the end of the pregnancy. Hence, as a treatment to be promoted for clinical use, a higher level of safety assessment with longer follow-up duration is necessary, for PRP introduces much greater concentrations of hematopoietic cells into the implantation environment that may have unknown detrimental effects on the embryo. In the circumstances, further studies are encouraged to assess the existing findings and clarify the medium- and long-term outcomes.

## Conclusion

Clinical studies for the efficacy of PRP in improving uterine endometrial function remain controversial. Most studies suggested that PRP treatment resulted in positive reproductive results in patients with thin endometrium, RIF, CE and AS. However, the best dosage and timing of PRP application remains unknown. Few studies assess the underlying mechanisms of the PRP treatment. PRP might improve the endometrial receptivity through the improvement of cell proliferation, vascularization, anti-inflammatory properties and the reduction in the degree of fibrosis, with the help of the concentrated peptides, GFs and cytokines in PRP. But for its potential clinical application, further explication of the mechanism is necessary. Though increasing evidence suggests the possible value of PRP treatment on the endometrium of infertile women, more carefully designed studies, especially RCTs, on larger scales are required. Further, the standard PRP preparation procedure and the strict indication of the PRP treatment should be published. Studies and follow-ups are needed on the long-term health and complications of the resulting child(ren).

## Author Contributions

YL drafted the manuscript. JQ and YS performed the critical revisions. All authors listed did contribute to the composing and review of the manuscript and reached an agreement on the final version.

## Funding

This study was supported by National Key R&D Program of China (No. 2019YFA0802604), the National Natural Science Foundation of China (No. 82130046, 81771648, 81901550), Shanghai leading talent program, Innovative research team of high-level local universities in Shanghai (No. SSMUZLCX20180401), Clinical Research Plan of SHDC (SHDC2020CR1046B), Shanghai Municipal Education Commission-Gaofeng Clinical Medicine Grant Support (No. 20161413), the orientation project to promote the National Natural Science Foundation of China in Renji Hospital affiliated to Shanghai Jiao Tong University School of Medicine (No. RJTJ-ZD-004), Shanghai Sailing Program (No. 19YF1428300), and Shanghai Commission of Science and Technology (No. 17DZ2271100).

## Conflict of Interest

The authors declare that the research was conducted in the absence of any commercial or financial relationships that could be construed as a potential conflict of interest.

## Publisher’s Note

All claims expressed in this article are solely those of the authors and do not necessarily represent those of their affiliated organizations, or those of the publisher, the editors and the reviewers. Any product that may be evaluated in this article, or claim that may be made by its manufacturer, is not guaranteed or endorsed by the publisher.
